# Severe pancreatic exocrine insufficiency presenting with hypoalbuminemia, anasarca, and pulmonary edema in a patient with prior gastrectomy and suspected immunoglobulin G4-related pancreatobiliary disease

**DOI:** 10.1007/s12328-026-02404-2

**Published:** 2026-07-23

**Authors:** Naoya Fujita, Kenichi Hashimoto, Akinori Sekizawa, Hiroshi Takazaki, Yosuke Ono, Yasuhiro Obuchi, Nobutaka Hirooka, Yuji Tanaka

**Affiliations:** 1 Division of Clinical Training Management, Medical Education and Clinical Training Center, National Defense Medical College, 3-2 Namiki, Tokorozawa, Saitama 359-8513 Japan; 2 Department of General Medicine, National Defense Medical College, 3-2 Namiki, Tokorozawa, Saitama 359-8513 Japan

**Keywords:** Pancreatic exocrine insufficiency, Immunoglobulin G4-related disease, Autoimmune pancreatitis, Gastrectomy, Hypoalbuminemia

## Abstract

A 55-year-old man with prior subtotal gastrectomy was admitted with generalized edema, 16-kg weight gain, dyspnea, and frequent watery pale stools. Laboratory tests revealed severe hypoalbuminemia (1.0 g/dL), anemia, elevated myogenic enzymes, liver dysfunction, and markedly decreased pancreatic enzyme levels. Computed tomography showed bilateral pleural effusion, pulmonary edema, ascites, diffuse soft tissue edema, and marked pancreatic atrophy. Stool Sudan III staining was positive, and urinary para-aminobenzoic acid excretion was reduced. Although cardiac, renal, and hepatic disorders were considered, they did not adequately explain the presentation. Pancreatic exocrine insufficiency was diagnosed. Serum immunoglobulin G4 was elevated at 199 mg/dL, and previous imaging showed poor visualization of the hilar bile duct, gallbladder wall thickening, and gallbladder atrophy. Therefore, possible immunoglobulin G4-related pancreatobiliary disease was considered the leading possible etiology of pancreatic atrophy and exocrine insufficiency, with prior gastrectomy as a modifying factor. Treatment with albumin, diuretics, and pancrelipase (1,800 mg/day) rapidly improved the overall condition of the patient. This case highlights that pancreatic exocrine insufficiency can present with severe third-space fluid shift, pulmonary edema, and compartment syndrome-like findings, highlighting the need to assess multifactorial causes.

## Introduction

Pancreatic exocrine insufficiency (PEI) is caused by reduced secretion or impaired activity of pancreatic digestive enzymes, leading to malabsorption of fats, proteins, and carbohydrates, and manifesting primarily as steatorrhea, weight loss, and nutritional deficiencies. Although chronic pancreatitis is the most common cause of PEI, a wide range of conditions can cause or contribute to it, including pancreatic malignancy, post-pancreatic surgery, and upper gastrointestinal surgery [1]. Among upper gastrointestinal procedures, gastrectomy is a well-known risk factor for PEI [2]. Autoimmune pancreatitis (AIP), a manifestation of immunoglobulin G4-related pancreatobiliary disease (IgG4-PBD), has increasingly been recognized as an important cause of PEI [3].

We report a case of PEI and diffuse pancreatic atrophy in a patient with a history of gastrectomy, in whom IgG4-PBD was considered a possible etiology. The patient demonstrated sequential changes over the preceding 7 years, including pancreatobiliary imaging abnormalities and subsequent elevation of serum IgG4 levels. The patient presented with severe hypoalbuminemia complicated by generalized edema, ascites, and pulmonary edema. Although the diagnosis was challenging, because differentiation from cardiac, renal, and hepatic diseases was required, the patient’s condition improved markedly following pancreatic enzyme replacement therapy. Through this case, we highlight the importance of considering PEI in patients presenting with unexplained generalized edema, and of evaluating possible structural pancreatobiliary disease, including IgG4-PBD, when PEI is accompanied by diffuse pancreatic atrophy.

## Case report

A 55-year-old man was admitted for evaluation of progressive generalized edema despite outpatient diuretic therapy. Lower extremity edema first appeared approximately 10 months before admission. Two weeks before admission, edema extended to the upper extremities and trunk. His body weight was 63 kg 1 year before admission, increased to 73 kg 10 days before admission, and further increased to 78 kg 2 days before admission. The patient reported watery, pale-colored stools occurring approximately 10 times per day starting approximately 10 days before hospitalization. He underwent subtotal gastrectomy for a duodenal ulcer 20 years before admission. In addition, 15 years before admission, the patient developed diabetes with reduced endogenous insulin secretion and had since been treated with intensive insulin therapy.

**Fig. 1:**
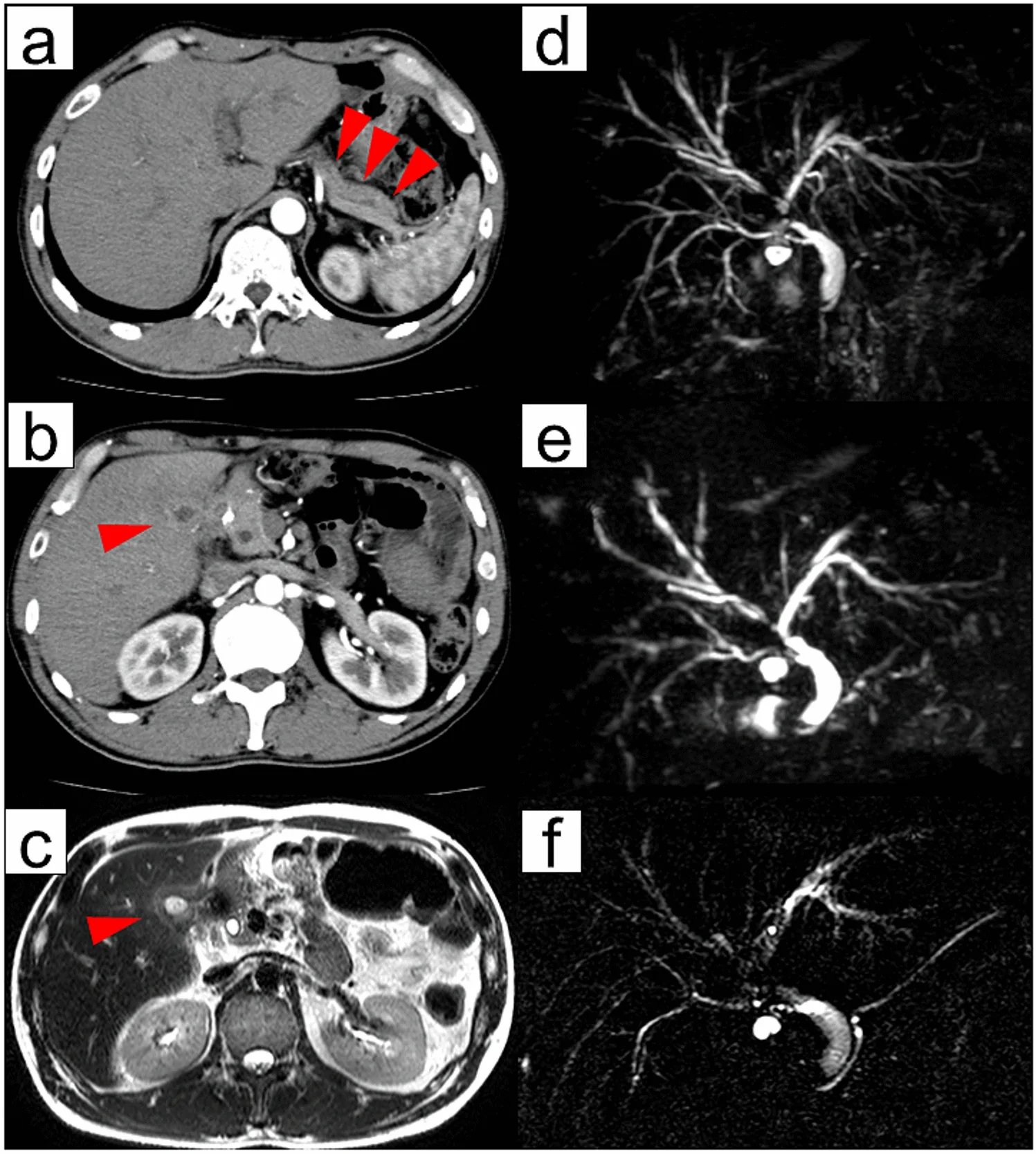
CT, MRI, and MRCP performed 7 years before admission. Mild atrophy of the pancreatic body and tail **(a)**, and gallbladder atrophy with circumferential wall thickening **(b)** are observed. The pericholecystic region shows T2 hyperintensity **(c)**, and poor visualization of the hilar bile duct was noted **(d)**. The previously poor visualization of the hilar region had improved on MRCP performed approximately 6 months after the initial imaging **(e)**. The main pancreatic duct was poorly visualized on three-dimensional MRCP, whereas its continuity was confirmed on two-dimensional imaging **(f)**. CT, computed tomography; MRI, magnetic resonance imaging; MRCP, magnetic resonance cholangiopancreatography; T2, T2-weighted image

Seven years before admission, the patient was referred to our hospital for evaluation of transient right upper abdominal pain and markedly elevated serum alkaline phosphatase and γ-glutamyl transpeptidase levels, which peaked at 5,685 U/L and 1,215 U/L, respectively. Computed tomography (CT) revealed gallbladder atrophy with wall thickening and mild atrophy of the pancreatic body and tail. No intraductal pancreatic stones or diffuse calcifications suggestive of chronic pancreatitis were identified. Magnetic resonance imaging (MRI) and magnetic resonance cholangiopancreatography (MRCP) revealed a T2-hyperintense signal surrounding the gallbladder, and the hilar bile duct was poorly visualized. The main pancreatic duct was poorly visualized on three-dimensional MRCP, whereas its continuity was confirmed on two-dimensional imaging. (Fig. 1a–f).

Gallbladder carcinoma was initially suspected and surgery was considered. However, positron emission tomography showed no abnormal uptake, biliary enzyme levels spontaneously normalized, the previously poor visualization of the hilar bile duct improved, and abdominal pain did not recur. Consequently, no further investigation or follow-up was undertaken. IgG4-related pancreatobiliary disease (IgG4-PBD) was also considered as a differential diagnosis; however, because the serum IgG4 level was 118 mg/dL, it was considered unlikely at that time. Regarding lifestyle habits, the patient reported daily alcohol consumption of approximately 1,000 mL of beer and approximately 180 mL of sake. He also had a smoking history of 20 cigarettes per day for 30 years.

On admission, the patient had a distended abdomen, and the lower extremities were markedly swollen. He complained of numbness of the feet and shortness of breath while standing and walking. His height was 166 cm, and body weight was 79 kg (body mass index, 28.7 kg/m^2^). Physical examination revealed a temperature of 36.0 ℃; blood pressure of 114/83 mmHg, pulse rate of 103 beats/min, respiratory rate of 16 breaths/min, and oxygen saturation of 94% on room air.

**Fig. 2:**
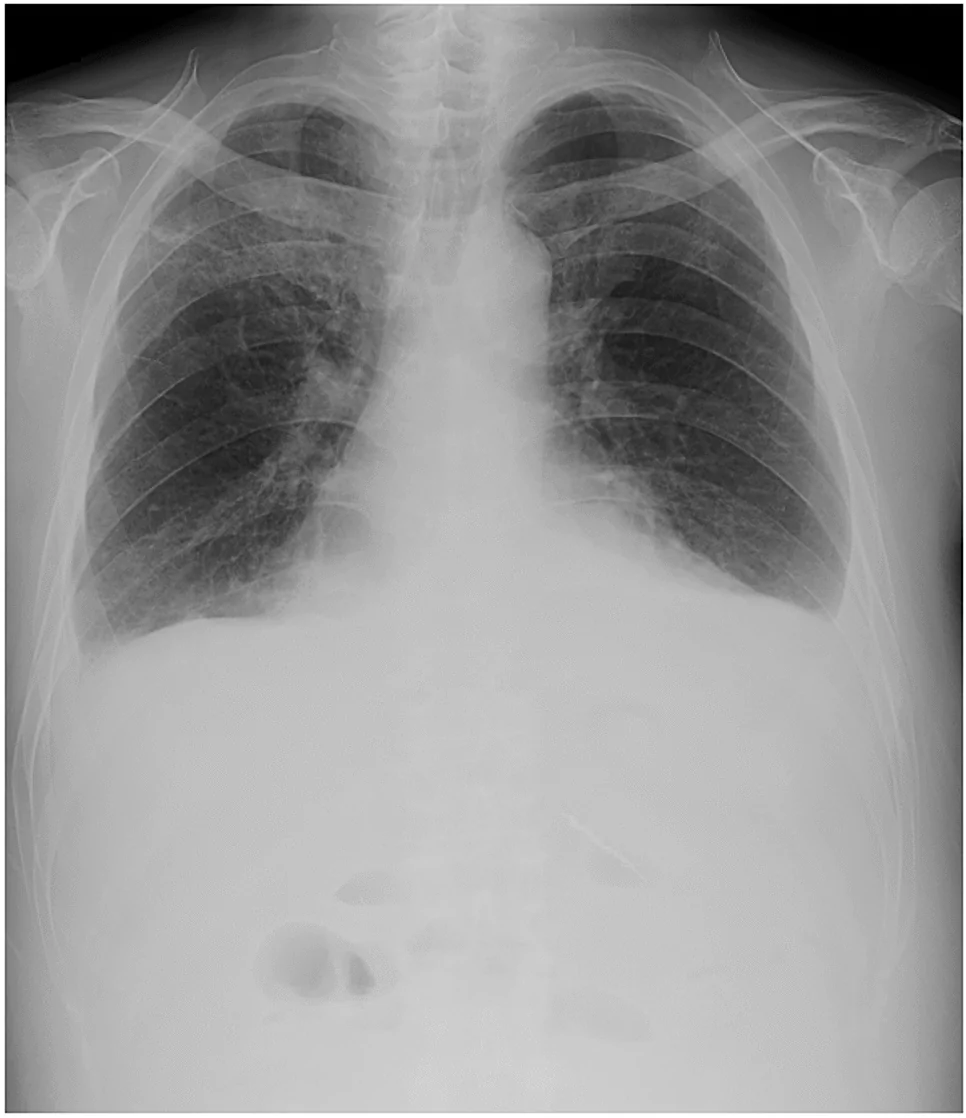
Admission chest radiograph showing bilateral pleural effusion and opacities in the right upper lung field

**Fig. 3:**
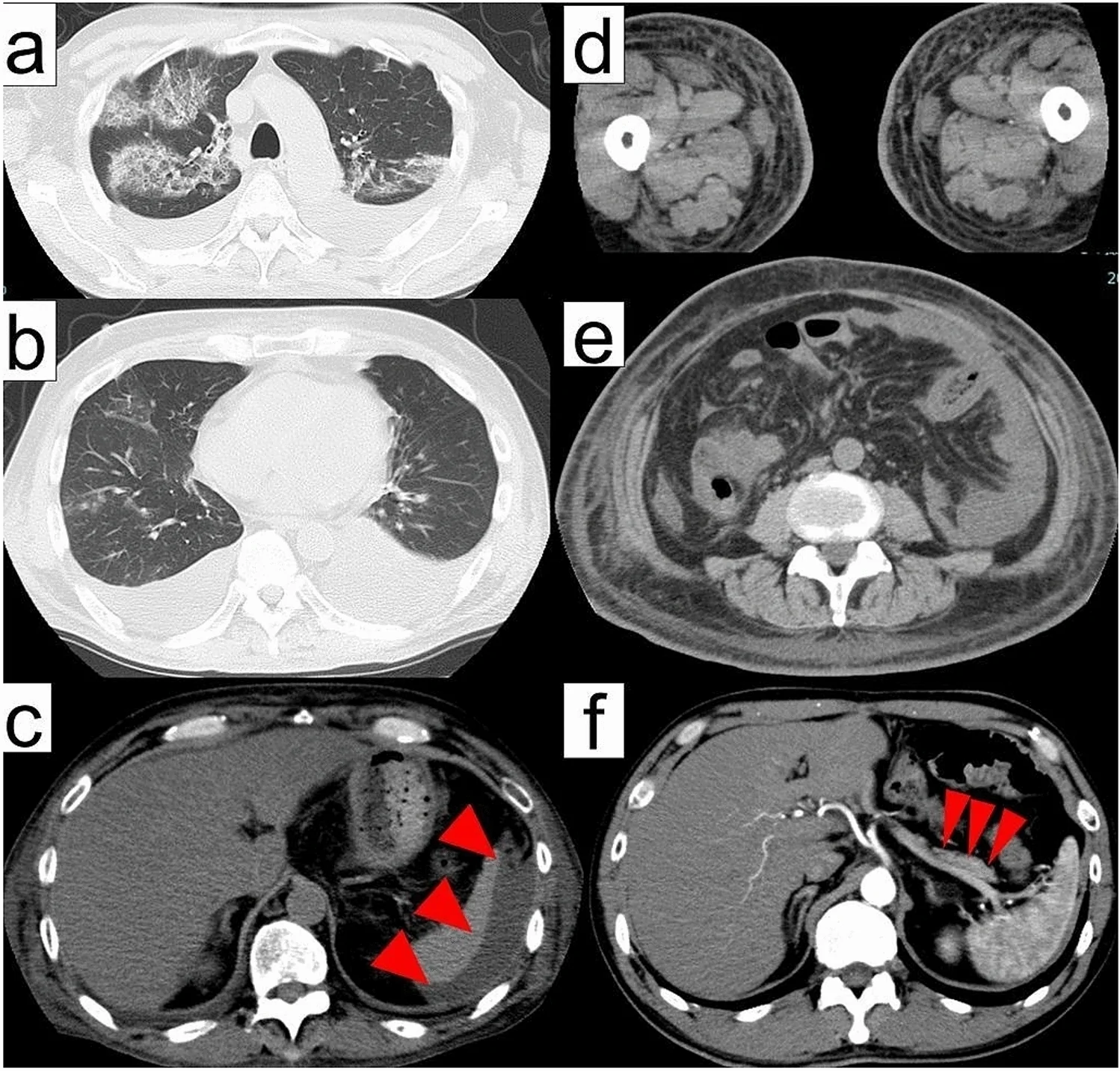
Admission CT showing bilateral pulmonary edema and pleural effusion (**a**, **b**), ascites (**c**), diffuse soft tissue and subcutaneous edema, including of the thighs (**d**, **e**), and upper abdominal postoperative pancreatic changes with marked pancreatic atrophy (**f**). CT, computed tomography

The laboratory findings on admission are shown in Table 1. The patient had severe hypoalbuminemia (1.0 g/dL), anemia (hemoglobin 7.9 g/dL), elevated myogenic enzymes, liver dysfunction, zinc deficiency, reduced coagulation activity, and markedly decreased pancreatic enzyme levels. The serum IgG4 level was elevated to 199 mg/dL. Brain natriuretic peptide was only mildly elevated (56.1 pg/mL), serum creatinine was normal (0.94 mg/dL), and urinalysis showed only trace protein. Chest radiography revealed bilateral pleural effusion and opacities in the right upper lung field (Fig. 2). CT showed bilateral pleural effusions, bilateral pulmonary edema, ascites, diffuse subcutaneous edema, and marked pancreatic atrophy. In addition, an anastomosis between the duodenum or jejunum and the residual stomach was identified, consistent with prior surgical reconstruction (Fig. 3a–f).

**Table 1:** Admission laboratory findings

WBC	4,000	/µL	T-Bil	0.4	mg/dL	Na	141	mmol/L
Hb	7.9	g/dL	AST	324	U/L	K	3.5	mmol/L
MCV	86	fL	ALT	151	U/L	Cl	111	mmol/L
Platelet count	18.0 × 10^4^	/µL	LDH	1193	U/L	Ca	6.8	mg/dL
PT activity	38.0	%	ALP	503	U/L	P	2.4	mg/dL
D-dimer	6.6	µg/mL	GGTP	77	U/L	Mg	1.6	mg/dL
			ChE	40	U/L	Zn	22	μg/dL
Fe	64	µg/dL	TP	5.2	g/dL	Cu	58	μg/dL
TIBC	Unmeasurable		Alb	1.0	g/dL	Hyaluronic acid	4,660	ng/mL
Ferritin	616.6	ng/mL	IgG	1,782	mg/dL	Type Ⅳ collagen	1,068	ng/mL
Vitamin B1	2.4	μg/dL	IgA	760	mg/dL	C-peptide	< 0.03	ng/mL
Vitamin B12	> 1,500	pg/mL	IgM	86	mg/dL	GADA	< 5.0	U/mL
Folic acid	6.2	ng/mL	IgG4	199	mg/dL			
			T-cho	54	mg/dL	***Urinalysis***		
TSH	10.40	μU/mL	BG	159	mg/dL	pH	5	
FT3	1.92	pg/mL	HbA1c	5.4	%	USG	1.013	
FT4	0.87	ng/dL	UA	8.0	mg/dL	Protein	( ±)	
BNP	56.1	pg/mL	BUN	6.0	mg/dL	Glucose	(-)	
			Cre	0.94	mg/dL	Blood	( ±)	
			eGFR	65.7	mL/min/1.73m^2^			
			CK	2,088	U/L			
			(BB 0%, MM 79%, MB 3%)					
			Amylase	26	U/L			
			P-amy	8	U/L			
			Lipase	3.7	U/L			
			PLA2	< 100	ng/dL			

Alb, albumin; ALP, alkaline phosphatase; ALT, alanine aminotransferase; AST, aspartate aminotransferase; BB, brain band; BG, blood glucose; BNP, brain natriuretic peptide; BUN, blood urea nitrogen; Ca, calcium; ChE, cholinesterase; CK, creatine kinase; Cl, chloride; Cre, creatinine; Cu, copper; eGFR, estimated glomerular filtration rate; Fe, iron; FT3, free tri-iodothyronine; FT4, free thyroxine; GADA, glutamic acid decarboxylase antibody; GGTP, γ-glutamyl transpeptidase; HbA1c, hemoglobin A1c; Ig, immunoglobulin; K, potassium; LDH, lactate dehydrogenase; MB, myocardial band; MCV, mean corpuscular volume; Mg, magnesium; MM, muscle band; Na, sodium; P, phosphorus; P-amy, pancreatic amylase; PLA2, pancreatic phospholipase A2; PT, prothrombin time; T-Bil, total bilirubin; T-cho, total cholesterol; TIBC, total iron binding capacity; TSH, thyroid stimulating hormone; UA, uric acid; USG, urine specific gravity; WBC, white blood cell; Zn, zinc

**Fig. 4:**
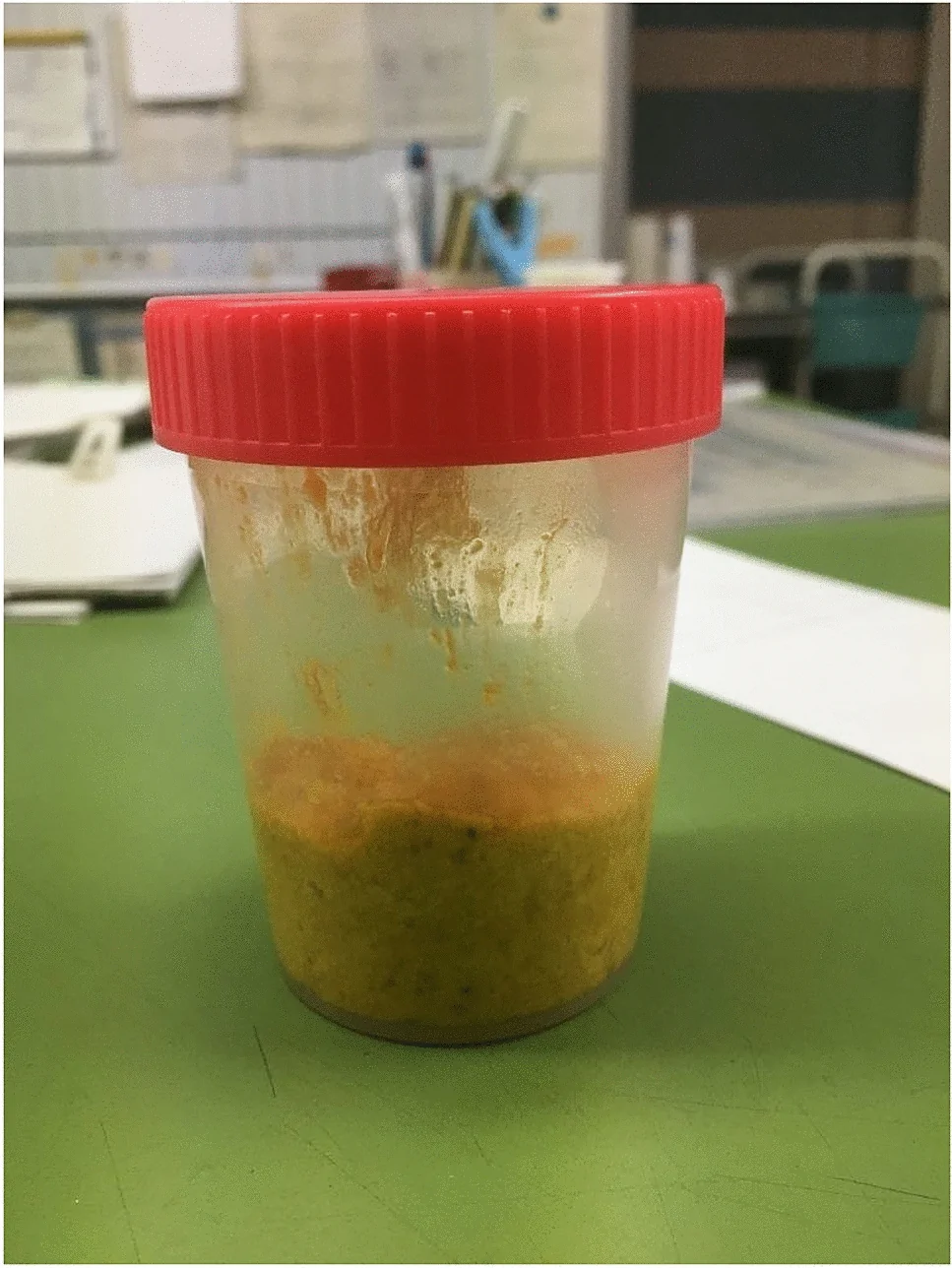
Gross appearance of the stool on admission. The stool is pale yellowish-white, with evidence of steatorrhea, supporting severe malabsorption

The stool was pale yellowish-white in appearance (Fig. 4), and stool samples were positive on Sudan III staining. The urinary para-aminobenzoic acid excretion rate in the pancreatic function diagnostic tests was 43.8%. This reduced rate, together with the imaging findings, low serum pancreatic enzyme levels, and severe malabsorptive phenotype, led to the diagnosis of PEI.

**Fig. 5:**
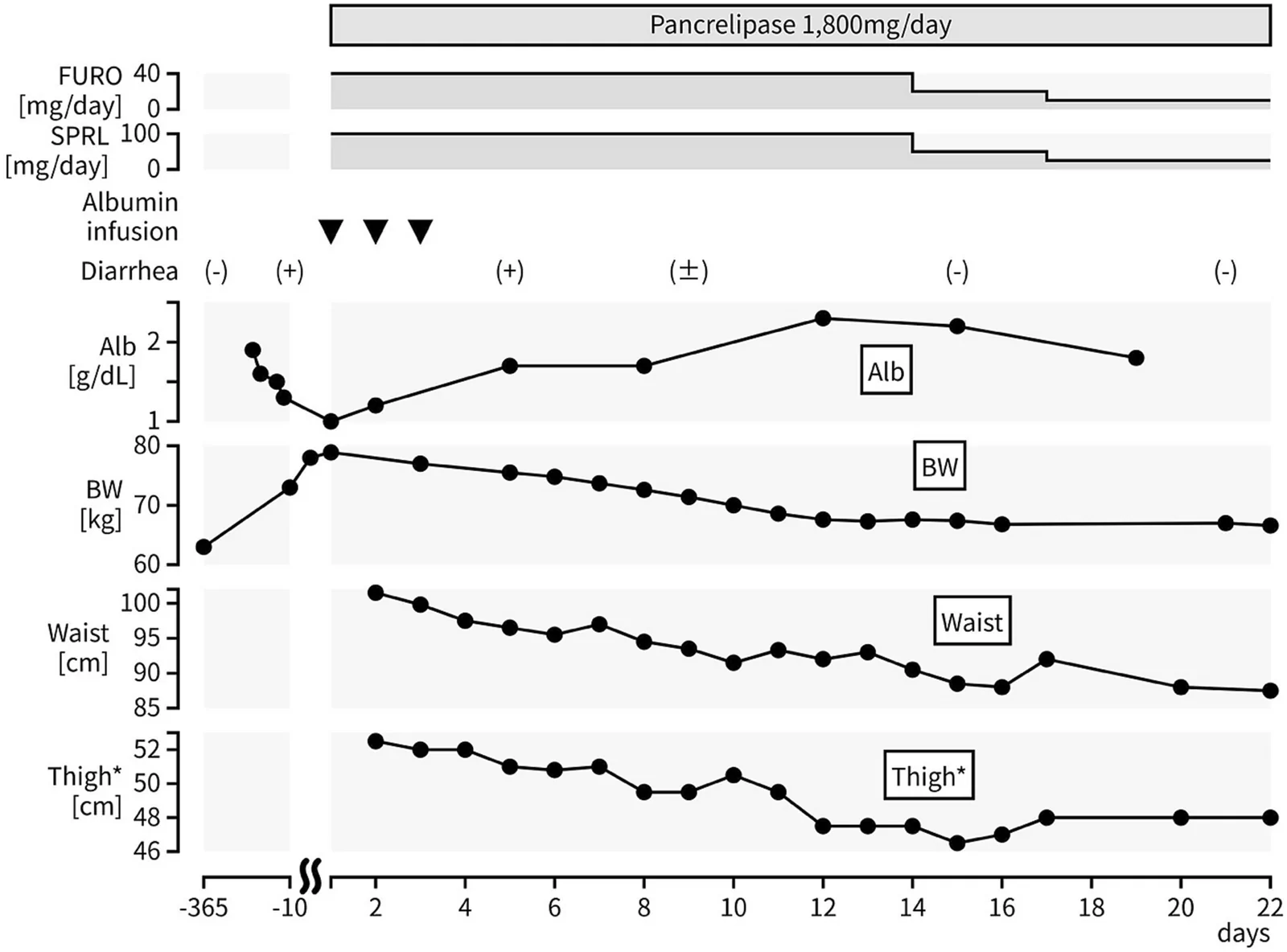
Clinical course from 1 year before admission to discharge. Body weight, waist circumference, and right thigh circumference decreased rapidly after treatment. BW, body weight; FURO, furosemide; HbA1c, hemoglobin A1c; SPRL, spironolactone

**Fig. 6:**
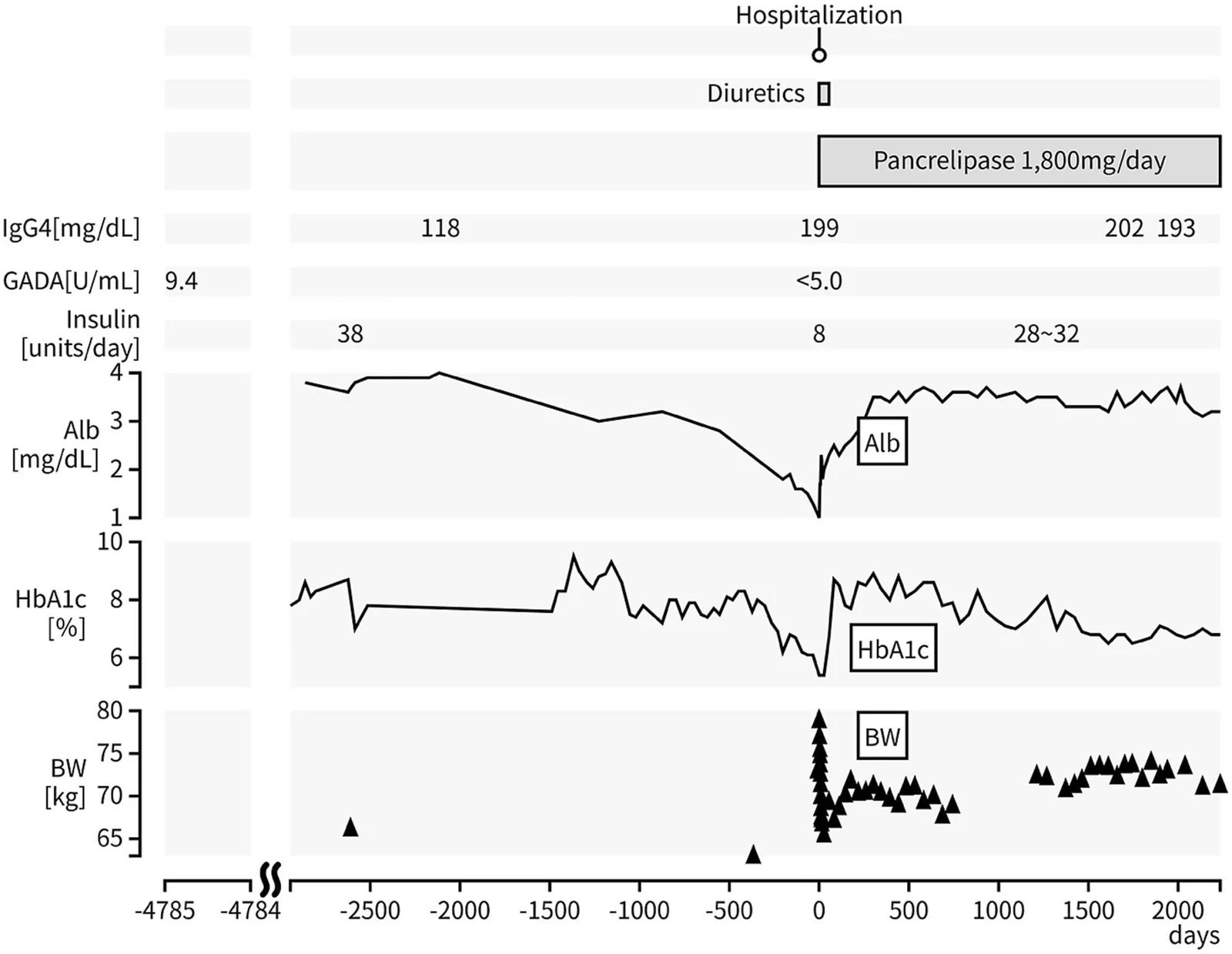
Approximately 20-year clinical course before and after hospitalization. Serum albumin levels gradually decreased, while body weight increased before admission. After hospitalization and the initiation of pancrelipase administration, the albumin level and body weight improved. Insulin requirements increased after nutritional recovery, consistent with improved nutrient absorption. Alb, albumin; BW, body weight; GADA, glutamic acid decarboxylase antibody; HbA1c, hemoglobin A1c; IgG4, immunoglobulin G4

The patient was treated with albumin infusion, diuretics, and oral pancrelipase (1,800 mg/day). His weight, waist circumference, and right thigh circumference decreased rapidly after therapy, and steatorrhea promptly improved. His generalized edema, dyspnea, and abdominal distension also improved. Pancrelipase administration resulted in elevated blood glucose levels, necessitating an increase in the daily insulin dose from 8 to approximately 25 units. The patient was discharged and has remained clinically stable during follow-up. The clinical course from 1 year before admission to discharge is shown in Fig. 5. The approximately 20-year clinical course before and after hospitalization is shown in Fig. 6.

## Discussion

This case highlights two important points. First, PEI may present not only as an isolated malabsorption syndrome but also with severe hypoalbuminemia, generalized edema, ascites, pulmonary edema, and elevated muscle-derived enzyme levels, which may lead to underrecognition. Second, IgG4-PBD should be considered as a possible etiology of PEI accompanied by marked pancreatic atrophy.

In the present case, the diagnosis of PEI was supported by watery pale stools, positive Sudan III staining, decreased bentiromide–para-aminobenzoic acid excretion, markedly low pancreatic enzyme levels, diffuse pancreatic atrophy on CT, and severe nutritional depletion. The prompt and sustained improvement after pancrelipase administration further supports this interpretation. The diagnostic challenge in the present case was that the dominant presentation was severe third-space fluid shift rather than chronic diarrhea alone. Severe hypoalbuminemia can cause diffuse edema and serous effusion. In this patient, pleural effusion, ascites, diffuse subcutaneous edema, pulmonary edema, and lower extremity induration initially broadened the differential diagnoses to include heart failure, renal disease, and hepatic disease. In particular, pulmonary edema suggested a cardiogenic or renal process; however, BNP elevation was only mild, renal function was preserved, and urinary protein was minimal. Additionally, overt hepatic failure did not adequately account for the clinical presentation. The overall clinical findings and prompt response to nutritional and enzyme replacement favored a PEI-related third-space fluid shift as the most consistent and plausible explanation. However, because a cardiac ultrasound examination was not performed during this hospitalization, we cannot completely rule out the possibility that transient cardiac dysfunction related to long-standing diabetes, smoking history, and chronic alcohol consumption contributed to the patient’s deteriorating condition.

We propose that superimposed and unrecognized AIP, supported by serial changes in imaging findings and serological data, led to progressive pancreatic parenchymal destruction that gradually depleted pancreatic reserve until a critical threshold was ultimately exceeded. PEI developed in 82% of patients with AIP at a median follow-up of approximately 6 years [4]. In a long-term follow-up study (mean follow-up 95.8 months, approximately 8 years) of patients with type 1 AIP receiving steroid therapy, Shimada et al. [5] reported that the mean pancreatic atrophy ratio compared with the presumed normal pancreatic volume was 59.3% ± 19.8%, with atrophy reaching as much as 86.5% (i.e., to as little as 13.5% of the baseline volume) in some patients with diffuse-type AIP or coexisting diabetes. This timeframe is broadly consistent with the interval between the initial suspicion of IgG4-related disease and the patient’s hospitalization in the present case. We suggest that the acute onset of steatorrhea approximately 10 days before admission, which coincided with the period of most rapid weight gain, may represent the clinical event at which this cumulative pancreatic reserve was ultimately exhausted. As no interval imaging was obtained between the evaluation 7 years before admission and the admission, the exact onset of diffuse pancreatic atrophy could not be determined.

To assess this possibility more rigorously, we evaluated the present findings against current diagnostic criteria for AIP and IgG4-related sclerosing cholangitis. The findings in this case only partially fulfilled these criteria [6–9]. At the time of the index episode 7 years before admission, the serum IgG4 level was 118 mg/dL, below the commonly applied cutoff of 135 mg/dL, and no pancreatic enlargement, capsule-like rim, or irregular narrowing of the main pancreatic duct was observed; mild atrophy of the pancreatic body and tail was the only pancreatic finding at that time. Biliary imaging demonstrated gallbladder wall thickening with pericholecystic T2 hyperintensity and poor visualization of the hilar bile duct, the latter of which spontaneously resolved on follow-up imaging approximately 6 months later. However, bile duct wall thickening and a characteristic cholangiographic stricture pattern, both required for a diagnosis of IgG4-related sclerosing cholangitis, were not documented. In addition, endoscopic or intraductal ultrasonography, which would have allowed direct assessment of bile duct wall thickness, was not performed. No histological specimen was obtained either at that time or during the current admission, and a therapeutic trial of steroids, which can support the diagnosis through a documented response, was never undertaken. The gallbladder findings are not included in the existing diagnostic criteria for IgG4-related sclerosing cholangitis, which focus on the bile duct rather than the gallbladder, and no formal diagnostic criteria currently exist for IgG4-related sclerosing cholecystitis.

Chronic pancreatitis was also considered in the differential diagnosis but was regarded as unlikely, because no pancreatic or intraductal calcifications, pancreatic stones, or main pancreatic duct dilation were observed on imaging obtained either 7 years before admission or at the current admission, and the patient had no history of recurrent epigastric pain suggestive of recurrent pancreatitis. The pattern observed in this case is atypical for ordinary chronic pancreatitis, which is usually accompanied by ductal dilation, stones, or calcification [10], and instead aligns with the atrophic changes described in the advanced natural history of AIP [11]. Collectively, several individual findings, including biliary imaging abnormalities, a subsequent rise in serum IgG4, and progressive pancreatic atrophy, are each compatible with antecedent IgG4-PBD; however, the absence of histological confirmation, a steroid trial, and fulfillment of formal imaging and serological thresholds at the time precluded, and continue to preclude, a definitive diagnosis according to current criteria. We therefore regard the evidence as suggestive of, rather than diagnostic of, antecedent AIP or IgG4-related sclerosing cholangitis.

The cumulative impact of altered gastrointestinal anatomy following gastrectomy on nutrient absorption is also considered a contributing factor to the clinical manifestation of PEI. The proposed mechanisms of PEI after gastrectomy include impaired vagal stimulation, altered gastrointestinal hormone signaling, rapid intestinal transit, poor coordination between meal delivery and biliopancreatic secretion, and abnormal mixing of nutrients with pancreatic juice [12]. However, if the prior gastrectomy were the primary cause of PEI, it would be difficult to explain why PEI did not become clinically apparent until more than 20 years after surgery. Although cases of PEI developing after a long interval following gastrectomy have been reported, to our knowledge, most have been limited to conference presentations in Japan, and such cases remain scarce [13, 14]. Several CT volumetry studies have reported that pancreatic atrophy can occur after gastrectomy. At 5 years postoperatively, the mean pancreatic volume was 67.4% after total gastrectomy with Roux-en-Y reconstruction and 70.6% after distal gastrectomy with Roux-en-Y reconstruction [15], and 82.9% (interquartile range: 73.6–90.2%) after distal gastrectomy with Billroth I reconstruction [16]. In the present case, CT imaging obtained 7 years before admission (approximately 13 years after surgery) showed only mild atrophy confined to the body and tail of the pancreas. Given these findings, we consider it unlikely that the diffuse pancreatic atrophy that developed over the subsequently 7 years can be attributed to gastrectomy alone.

The risk of post-gastrectomy PEI has been reported to differ by reconstruction type, with greater disruption of the coordinated postprandial release of pancreatic enzymes after reconstructions that bypass the duodenum, such as Billroth II or Roux-en-Y, than after Billroth I reconstruction [17, 18]. The operative records could not be obtained in this patient, and although contrast-enhanced CT at the current admission demonstrated an anastomosis between the residual stomach and the duodenum or jejunum, it was not possible to distinguish between Billroth-type and Roux-en-Y reconstruction based on imaging alone. Regardless of the specific reconstruction performed, however, the approximately 20-year interval between surgery and symptom onset in the present case is among the longest previously documented, and the abrupt onset and severity of malabsorption and third-space fluid shift observed here indicate that gastrectomy alone does not sufficiently explain the clinical presentation, further supporting the interpretation that an additional structural process, such as IgG4-PBD, was required.

Approximately half of patients with AIP present with concurrent exocrine and endocrine insufficiency at the time of diagnosis [3]. In this case, however, diabetes has already been diagnosed before the onset of PEI, a finding that warrants further consideration. A review of past medical records has revealed that glutamic acid decarboxylase antibody was positive (9.4 U/mL; reference value ≤ 1.4 U/mL) 13 years before the current hospitalization. This suggests that the etiology of diabetes in this patient was type 1 from the outset and likely represented a condition independent of type 3c (pancreatogenic) diabetes associated with AIP [19]. At admission, the glutamic acid decarboxylase antibody was negative (< 5.0 U/mL), consistent with the well-recognized progressive disappearance of islet autoantibodies over the long-term course of type 1 diabetes [20]. Collectively, this case may represent a relatively rare clinical entity in which type 1 diabetes and AIP-related PEI coexisted.

Three potential mechanisms may explain the liver dysfunction observed at admission. First, malnutrition-related fatty liver secondary to PEI [21]. In this case, hepatic function improved rapidly following the initiation of pancreatic enzyme replacement therapy and nutritional support without direct hepatological intervention, a finding highly consistent with a nutritional etiology. Second, metabolic dysfunction-associated steatotic liver disease in the setting of exogenous insulin therapy. Hyperinsulinemia, whether endogenous or exogenous, promotes hepatic de novo lipogenesis via activation of sterol regulatory element-binding protein-1c, thereby contributing to hepatic steatosis [22]. Therefore, metabolic dysfunction-associated steatotic liver disease related to intensive insulin therapy cannot be excluded. Third, alcoholic fatty liver disease. The patient reported an ethanol intake of approximately 62 g per day (with possible underreporting). Furthermore, the persistent aspartate aminotransferase-predominant liver enzyme pattern (aspartate aminotransferase 324 U/L > alanine aminotransferase 151 U/L) and elevated γ-glutamyl transpeptidase levels (77 U/L) at admission, together with the fact that the patient continued to consume the same amount of alcohol after discharge as before admission, with persistently abnormal laboratory findings, collectively suggest that chronic alcohol intake may have contributed to hepatic steatosis.

In conclusion, clinicians should be aware of the possibility of PEI in post-gastrectomy patients presenting with otherwise unexplained hypoalbuminemia, generalized edema, or pulmonary involvement. In this case, we consider IgG4-PBD, manifesting as antecedent AIP, to represent the primary pathophysiological driver of progressive pancreatic atrophy and subsequent PEI, with prior gastrectomy, chronic alcohol consumption, and diabetes mellitus acting as modifying or aggravating factors rather than independent primary causes. This hierarchical framework does not exclude the possibility that these factors interacted with one another, and their relative contributions and temporal relationships should be carefully assessed in similar cases. A comprehensive and integrated analysis of the pathophysiology, rather than attribution to a single cause, is essential to accurately understand each patient’s clinical condition.
